# High dose antipsychotic treatment monitoring audit

**DOI:** 10.1192/bjo.2021.912

**Published:** 2021-06-18

**Authors:** Jake Scott, Jose Belda

**Affiliations:** Sussex Partnership NHS Foundation Trust

## Abstract

**Aims:**

To quantify how many patients were prescribed high dose antipsychotic treatment (HDAT) and establish whether guidance for monitoring HDAT was being followed in an Assertive Outreach Team.

**Background:**

Severe mental health disorders are associated with significant premature mortality, predominantly due to physical health conditions. Antipsychotic medications are associated with side effects, including metabolic syndrome and QT prolongation, which increase the risk of serious physical illness. HDAT is defined as when the total dose of antipsychotics prescribed exceeds 100% of the maximum BNF dose, if each dose is expressed a percentage of its maximum dose. There is limited evidence of clinical benefit with HDAT but an increased risk of side effects. Patients prescribed HDAT should therefore be monitored for side effects and clinical benefit. Sussex Partnership NHS Foundation Trust developed a form specifically for this purpose, to be completed in addition to a physical health assessment.

**Method:**

All patients on caseload were audited using the electronic notes. Current inpatients were excluded, as inpatient HDAT monitoring forms are attached to paper drug charts and therefore were not available for review.

**Result:**

A total of 61 patients were audited. Nine were excluded due to being inpatients. 16 were on community treatment orders and 26 were prescribed a long-acting antipsychotic injection. 10 were prescribed clozapine. The median number of medications prescribed was one. Four patients were prescribed HDAT ranging from 117-150% of the maximum BNF dose. Of these four, one had a HDAT form but this was out of date. 39 of 52 (75%) patients audited had had a physical health assessment in the past 12 months. Two of the 13 missing a physical health assessment were on HDAT.

**Conclusion:**

Physical health monitoring should be carried out for all patients on antipsychotics, but is particularly important for patients on HDAT. This audit identified a problem in both general physical health checks and HDAT monitoring. On discussion with the multi-disciplinary team a number of barriers to appropriate physical health monitoring were identified. There was a lack of awareness within the multi-disciplinary team that patients were receiving HDAT and regarding the implications for side effects. A reliable system to highlight the need for physical health checks was also missing and the team did not have sufficient equipment to perform the necessary checks. Identifying these barriers should enable improvements in physical health and HDAT monitoring which can be re-audited.

